# Cancer screening risk literacy of physicians in training: An experimental study

**DOI:** 10.1371/journal.pone.0218821

**Published:** 2019-07-03

**Authors:** Dafina Petrova, Guiliana Mas, Gorka Navarrete, Tania Tello Rodriguez, Pedro J. Ortiz, Rocio Garcia-Retamero

**Affiliations:** 1 Cancer Registry of Granada, Andalusian School of Public Health, Granada, Spain; 2 Instituto de Investigación Biosanitaria ibs.Granada, University of Granada, Granada, Spain; 3 CIBER of Epidemiology and Public Health (CIBERESP), Madrid, Spain; 4 Mind, Brain, and Behavior Research Center, University of Granada, Granada, Spain; 5 School of Medicine, Instituto de Gerontología, Universidad Peruana Cayetano Heredia, Lima, Perú; 6 Center for Social and Cognitive Neuroscience (CSCN), School of Psychology, Universidad Adolfo Ibáñez, Santiago de Chile, Chile; 7 Harding Center for Risk Literacy, Berlin, Germany; Middlesex University, UNITED KINGDOM

## Abstract

We investigated what factors may foster or hinder physicians’ cancer screening risk literacy–specifically the ability to understand evidence regarding screening effectiveness and make evidence-based recommendations to patients. In an experiment, physicians in training (interns and residents) read statistical information about outcomes from screening for cancer, and had to decide whether to recommend it to a patient. We manipulated the effectiveness of the screening (effective vs. ineffective at reducing mortality) and the demand of the patient to get screened (demand vs. no demand). We assessed participants’ comprehension of the presented evidence and recommendation to the patient, as well as a-priori screening beliefs (e.g., that screening is always a good choice), numeracy, science literacy, knowledge of screening statistics, statistical education, and demographics. Stronger positive a-priori screening beliefs, lower knowledge of screening statistics, and lower numeracy were related to worse comprehension of the evidence. Physicians recommended against the ineffective screening but only if they showed good comprehension of the evidence. Physicians’ recommendations were further based on the perceived benefits from screening but not on perceived harms, nor the patient’s demands. The current study demonstrates that comprehension of cancer screening statistics and the ability to infer the potential benefits for patients are essential for evidence-based recommendations. However, strong beliefs in favor of screening fostered by promotion campaigns may influence how physicians evaluate evidence about specific screenings. Fostering physician numeracy skills could help counteract such biases and provide evidence-based recommendations to patients.

## Introduction

Many decisions about health involve the consideration of complex numerical information about risks and benefits. On such occasions, medical professionals are expected to be *risk literate* decision makers and advisors to their patients [1–3]. *Risk literacy* broadly refers to one's practical ability to evaluate and understand risk in the context of informed decision making: for instance, to understand the benefits and harms of available treatments and to be able to make informed, value-consistent decisions based on the information at hand [4]. Risk literacy is closely related to statistical literacy, which refers to physicians’ ability to understand the terminology and statistical aspects associated with the design, analysis, and conclusions of original research [1,5]. Thus, when physicians are required to make recommendations to patients based on research evidence, some statistical literacy would be required to understand the relevant evidence.

Risk literacy is essential for practicing evidence-based medicine and facilitating shared decision making of patients, because it enables medical professionals to (i) understand what the net benefits of potential treatments are, (ii) communicate accurate information to patients, and (iii) make evidence-based recommendations. Whereas much is known about the challenges faced by patients in decisions involving risk information [6], fewer studies have examined risk literacy in physicians and its implications for recommendations to patients.

Illustrative examples of the importance of risk literacy are cancer screening controversies and the documented difficulties to understand the associated evidence [3,7–9]. For instance, a representative survey of US primary care physicians showed that physicians were strongly influenced by irrelevant evidence in their endorsement of screening tests [10]. Many physicians mistakenly thought that increased detection and better survival rates demonstrate that screening saves lives (47% and 76% of physicians, respectively) [10]. This shows that the majority of physicians were not aware that these indicators are influenced by both lead-time and overdiagnosis biases, and that their improvement is not sufficient to demonstrate screening effectiveness [11]. Whereas increased detection is a goal of cancer screening, for screening to be actually effective, it must lead to a reduction in mortality rates (and not 5-year survival rates) compared to a situation without screening. In lead-time bias, 5-year survival rates (the percentage of patients alive 5 years after diagnosis) are inflated by earlier diagnosis in the screening group even if mortality is equal across groups; in overdiagnosis bias, survival rates are inflated by the detection of nonprogressive cancers even if mortality is equal across groups [10]. In addition, besides benefits, cancer screening can also have harms, such as false positive tests followed by unnecessary and anxiety-provoking biopsies, and overdiagnosis and unnecessary treatment due to the detection of nonprogressive cancers [12]. To fully appreciate the benefits and harms of screening and advise their patients, physicians need to understand the associated statistical evidence. However, a recent study demonstrated that physicians’ inability to understand statistical evidence regarding screening effectiveness can lead to biased and incomplete communication to patients regarding screening, in which important harms are omitted [13].

In this research, we aimed to identify factors that can facilitate or hinder informed decision making in the context of cancer screening. Physicians’ recommendations may be influenced by factors such as the extent of life-saving benefits the particular screening offers, the physicians’ specific skills or beliefs about screening, or the patient’s demand for screening. In the current study, we investigated how these factors influence physicians’ comprehension of evidence regarding screening effectiveness and screening recommendations, and discuss the results in the context of improving general physician risk literacy.

### What factors can influence comprehension?

#### Beliefs about screening

National surveys have shown that the public generally views cancer screening very positively. People tend to think that screening is always a good choice and overestimate the benefits of some cancer screenings by at least tenfold [8,14,15]. Such beliefs are often reinforced by screening campaigns that fail to specify the extent of benefit and important risks like false positive tests or overdiagnosis [16]. Such campaigns may create the impression that cancer screening is always the best choice and not a matter of careful evaluation of the evidence of benefits and harms. Importantly, recent research showed that strong positive beliefs about the general goodness of screening, like the ones discussed above, can have detrimental effects on patients’ decision making [9,17]. For instance, upon reading information about screening benefits and harms, participants who had stronger positive beliefs about screening were more likely to want to get screened, even when the screening offered no benefits and could cause substantial harms [17].

However, it is not clear to what extent beliefs would influence physicians’ evaluation of the evidence. Research from psychology has shown that people tend to discount or evaluate more shallowly evidence that is contrary to their existing beliefs (i.e., motivated reasoning; [18]). Similarly, once a belief has been established in people’s minds (i.e., screenings are always life-saving), it may be especially difficult to correct it in light of new evidence [19]. Hence, it is reasonable to hypothesize that physicians who have strong positive beliefs about screening will show less accurate comprehension of the evidence compared to those who do not share these beliefs. This could be due to not investing enough effort to understand the evidence (because of already existing convictions) or discounting the evidence contrary to their beliefs as invalid or misunderstood. Alternatively, if beliefs are not related to comprehension, that would indicate that physicians’ extensive training can protect them from common psychological biases often found in laypersons.

#### Specific physician competencies

Although one might expect that physicians’ extensive education prepares them to understand complex statistical evidence and make evidence-based recommendations, research shows that physicians vary strongly in their abilities [20–24]. For instance, statistical numeracy, from here on referred to as numeracy for short-the practical ability to understand expressions of risk and probability-is a robust predictor of medical decision making of both patients and medical professionals across diverse contexts [4,6,25–27]. Compared to physicians with high numeracy, physicians with lower numeracy are more likely to misunderstand risk reduction information [21], more likely to make incorrect diagnostic inferences from screening tests [24], and misunderstand the risks of post-surgical side effects [22].

Besides numeracy, physicians’ science literacy may also contribute to physicians’ comprehension of evidence regarding screening effectiveness. Science literacy refers to basic knowledge about how science generates and assesses evidence [28,29]. Whereas most physicians are expected to have high levels of science literacy, low science literacy, even among a minority of physicians could have serious negative consequences for their comprehension and decisions. For instance, in the context of screening, it is essential to know that a comparison with a control group (without screening) is necessary to assess the benefits and harms attributable to the screening.

Finally, the specific knowledge of what screening statistics are relevant for determining if screening is effective or not should also help physicians understand and properly evaluate screening effectiveness [10,13]. As mentioned above, misconceptions that detection rates and 5-year survival rates are sufficient to demonstrate that screening saves lives may lead to wrong inferences about the effectiveness of screening.

### What factors influence physicians’ recommendations?

#### Comprehension of the evidence

Comprehension of cancer screening outcomes–the ability to interpret the evidence for screening benefits and harms and derive plausible risk estimates regarding patient outcomes–is essential, because it can influence perceptions of benefits and harms and hence decisions about screening. For instance, recent research using path modeling showed that laypersons’ comprehension of the statistical evidence regarding screening effectiveness influenced perceived benefits (but not perceived harms) of screening, which in turn were related to intentions to undergo screening [9,17]. In the current research, we investigate to what extent this model obtained from laypersons’ judgments about screening generalizes to physicians. In particular, given screenings with either small or non-existent benefits, we expect better comprehension of the evidence to be related to smaller perceived benefits and weaker recommendations for screening [9,17]. Conversely, physicians who misunderstand the evidence would be more likely to recommend screening, even when it has no benefits.

#### Screening effectiveness

Cancer screenings vary strongly in the degree of benefits and harms depending on the cancer, procedure, or age of the person being screened [30]. For instance, although breast cancer screening with mammography is associated with certain harms, experts generally conclude that it is effective (i.e, life-saving) for women of certain ages [31]. In contrast, screening for prostate cancer with PSA tests is associated to similar harms but its benefits were judged to be negligible by experts, deeming it ineffective for most age-groups [32]. To the extent that physicians aim that their recommendations are evidence-based, one would expect physicians to recommend a screening that is effective (i.e., reduces mortality) and recommend against a screening that is not effective (e.g., does not reduce mortality despite detecting more cancers). This may, however, strongly depend on physicians’ ability to understand the evidence about screening effectiveness: physicians who have low comprehension of the evidence may be equally likely to recommend effective and ineffective screening tests.

#### Patient demand

Another factor that could influence physicians’ recommendations is patient demand (i.e., the wish of the patient to attend screening or not). For example, some physicians practice defensive decision making–they recommend treatments they would not choose themselves for fear of legal prosecution [33]. Research shows many physicians order screening for their patients although they do not believe that it is life-saving, and they do so because of strong patient demand, fear of lawsuits, or the belief that it represents a standard of practice [34–36]. Outside the context of cancer screening, more recent evidence shows that patient demand for antibiotics also results in more antibiotics prescriptions [37]. Overall, we expect patient demand to increase physicians’ screening recommendations.

#### The current research

For the current research we recruited physicians in training and presented them with a hypothetical case of a patient who asked for advice regarding cancer screening. To test our hypotheses, we experimentally manipulated the effectiveness of the screening: effective (only moderately) vs. ineffective, and the demand of the patient for screening: demanding screening vs. not demanding it. Physicians were randomly assigned to one of the resulting four versions.

To summarize our hypotheses, regarding comprehension of the evidence, we expected that less positive beliefs about screening, higher numeracy, higher science literacy, and better knowledge of screening statistics would be related to better comprehension of the evidence. We expected that patient demand would increase recommendations. In contrast, we expected that screening *in*effectiveness would *de*crease recommendations; however, only among physicians who had good comprehension (i.e., an interaction between effectiveness and comprehension), as physicians with low comprehension may mistakenly recommend the ineffective screening.

Regarding the role of perceived benefits and harms of screening, and having in mind the evidence we presented to participants (i.e., screenings with small or inexistent benefits), we expected that better comprehension of the evidence would be related to smaller perceived benefits and larger perceived harms, but that perceived benefits would be a stronger predictor of recommendations compared to perceived harms, as found in previous research with laypersons [9,17].

## Method

### Participants and procedure

Participants were physicians in training from the Cayetano Heredia University in Lima (Peru) who were doing clinical rotation in the internal medicine wards of the Arzobispo Loayza y Cayetano Heredia hospitals in Lima. The Cayetano Heredia University has one of the top-ranking programs in medicine in Peru and Latin America. The population of interest consisted of 429 physicians registered that year (128 6^th^ year students, 95 interns, and 206 residents). For the duration of the study we approached 173 (40%) potential participants (average age = 28 years, SD = 4.8, 53% female) and all agreed to participate. The majority of participants (N = 119, 68%) were residents representing a variety of 14 sub-specialties (e.g., nephrology, hematology, internal medicine, respiratory medicine, family medicine, oncology, etc.). Thirty-nine (23%) participants were advanced medical students in their 6^th^ year and 15 (9%) were in their 7^th^year (interns).

Participants signed an informed consent and filled in a paper-and-pencil questionnaire. All instruments were in Spanish. The questionnaire started with demographic questions and assessment of a-priori screening beliefs. Participants then read a randomly assigned version of the screening scenario described below and answered questions about it. The questionnaire ended with an assessment of numeracy and science literacy. Ethical approval was obtained from the Ethics Committee of the Cayetano Heredia University in Lima and data was collected in October and November 2015.

### Materials and measures

#### Demographics and experience

Participants indicated their age, gender, and stage of academic training (6^th^ year, 7^th^ year or resident) and academic specialty, if relevant. Participants indicated if they had taken a course in research methodology and/or statistics (yes/no) and if they had published a scientific study in an indexed journal (yes/no).

#### A-priori positive screening beliefs

This was measured with a questionnaire from Petrova et al. [17], Cronbach’s α = .80, on a separate page and before the screening scenario described below was introduced. On scales from 1 (strongly disagree) to 7 (strongly agree), participants indicated to what extent they agreed with 5 statements that reflected positive attitudes towards screenings in general, for instance for diseases such as cancer: “Participating in screening always has more advantages than disadvantages”, “Screening cannot hurt anyone”, “It is always better to participate in screening”, “If one has the opportunity, one should always participate in screening”, and “Foregoing screening is irresponsible”). The final score was a sum of all items ranging from 5 to 35, where a higher score indicates more positive a-priori screening beliefs.

#### Screening scenario

Participants were asked to imagine that they were practicing physicians and that a 55-year-old patient had come to ask them about screening for cancer X. They were about to read some information about the screening and consider whether to recommend it to the patient. No specific cancer was mentioned to avoid the influence of participants’ knowledge about existing cancer screening programs (see [13] for a similar procedure). In the screening scenario we experimentally manipulated the demand of the patient to get screened (demand vs. no demand) and the effectiveness of screening at reducing mortality (effective vs. ineffective). Participants were randomly assigned to one of the resulting four versions of the scenario.

Patient demand: Half of the participants read that the patient had a lot of information regarding cancer X and the screening from the media, friends, and family. He was also very worried about cancer X and wanted to get screened; nevertheless, he wanted to ask his physician’s opinion (demand condition). The other half of participants read that the patient had little information about cancer X and the screening and was hence undecided about getting screened and wanted to ask his physician’s opinion (no demand condition).

**Fig 1:**
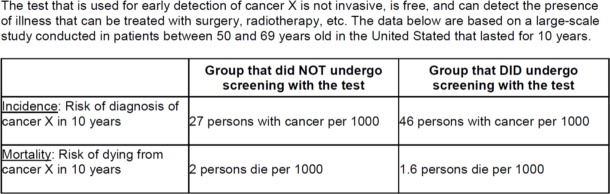
Information shown to participants regarding detection and mortality from cancer X with and without screening. The information was based on outcomes from the European Randomized Study of Screening for Prostate Cancer [38] as shown in [10]. The information depicted is from the effective condition. In the ineffective condition, participants saw the same information with the exception that mortality with screening was kept equal to mortality without screening (= 2 persons per 1000). Effectiveness is demonstrated by a significant reduction in mortality in the screening group compared to the group without screening. Harms are implied by the much larger detection of cancer in the screening group but only modest (in the effective condition) or nonexistent (in the ineffective condition) reduction in mortality. These data suggest that many patients are potentially overdiagnosed and treated unnecessarily.

Screening effectiveness: Half of participants read statistics showing that screening was modestly effective at reducing mortality (effective). The other half read that screening was not life-saving (ineffective). The exact information provided and further explanation is included in Fig 1.

#### Knowledge of screening statistics

It was measured with four questions adapted from Wegwarth et al. [10] and Petrova et al. [9], which assessed participants’ knowledge of what statistics are relevant for determining if screening is effective or not (see Table 1). Each question was scored as correct (1) or incorrect (0) and the final score was a sum of the number of correct answers (0–4).

**Table 1:** Items used to assess knowledge of screening statistics and comprehension of the evidence based on Wegwarth et al. [10] and Petrova et al. [9].

Item text	N (%) correct overall (N = 172)	N (%) correct in the ineffective condition (N = 87)	N (%) correct in the effective condition (N = 85)
**Knowledge of screening statistics**			
What demonstrates that a screening test saves lives? (a-c) Q1. a) Cancers detected due to screening have better 5-year survival rates compared to cancers detected due to symptoms. *_____Yes*, *it demonstrates*. *__X__No*, *it does not demonstrate*. *_____I don’t know*.	28 (16%)	16 (18%)	12 (14%)
Q2. b) More cancers are detected in screened populations than in unscreened populations. *_____Yes*, *it demonstrates*. *__X__No*, *it does not demonstrate*. *_____I don’t know*.	95 (55%)	48 (55%)	47 (55%)
Q3. c) In a randomized trial, mortality rates are lower in the screening group than in the group without screening. *__X__Yes*, *it demonstrates*. *_____No*, *it does not demonstrate*. *_____I don’t know*.	124 (72%)	65 (75%)	58 (68%)
Q4. To know whether a screening test saves lives, we need to compare the survival rates of the two groups after 5 years. *______true* *__X___false*	58 (34%)	29 (33%)	29 (34%)
**Comprehension of the evidence regarding screening for cancer X**			
Q1. The screening test for cancer X saves lives. ___X__true* ___X__false* ______it is not possible to tell from the available data *True in the effective condition and false in the ineffective condition.	86 (50%)	47 (54%)	39 (46%)
Q2. Imagine a group of 2000 people between 50 and 69 years old who participate in regular screening for the next 10 years, and another similar group of 2000 people who do not participate in screening. How many fewer persons would die from cancer X in the group with screening compared to the group with screening? ______ deaths fewer out of 2000* *0 in the ineffective condition and between 0 and 1 in the effective condition.	73 (42%)	29 (33%)	44 (52%)^a^
Q3. According to the data, some people may have been diagnosed and treated for cancer X unnecessarily. *___X__true* *______false*	59 (34%)	28 (32%)	30 (35%)
4. People in the screening group must have had more risk factors associated with cancer X compared to the group without screening. *______true* *___X__false*	129 (75%)	65 (75%)	63 (74%)
Q5. After 10 years, 19 people in the screening group are alive thanks to screening. *______true* *___X__false*	113 (65%)	55 (63%)	57 (67%)

Correct answers are marked with an X.

^a^Significant difference between the ineffective and the effective condition according to chi-square test, p < .05.

#### Comprehension

It was measured with five questions adapted from Petrova et al. [9] that measured participants’ comprehension of the presented evidence regarding screening for cancer X (i.e., their ability to interpret and derive risk estimates based on the depicted results (see Table 1)). Each question was scored as correct (1) or incorrect (0) and the final score was a sum of the number of correct answers (0–5).

#### Perceived benefits and harms

Participants had to indicate how they would describe the (a) benefits and (b) harms produced by the screening for cancer X on scales from (1) *none* to (6) *very large*.

#### Recommendation

Participants indicated if they would recommend the screening to their hypothetical patient on scales from (1) *definitely not* to (6) *definitely yes*.

#### Numeracy

It was measured with the Berlin Numeracy Test-Schwartz (BNT-S) following Cokely et al. [25]; see RiskLiteracy.org. The test has been validated for use in medical professionals and consists of 7 items of varying difficulty: three items from Schwartz et al. [39] and 4 items from the Berlin Numeracy Test; e.g., “Imagine that we are throwing a five-sided die 50 times. On average, out of these 50 throws how many times would this five-sided die show an odd number (1, 3 or 5)?” Our choice of the combined BNT-S test was based on previous work in this population showing that the combinations of easier (Schwarz) and more difficult items (BNT) would show better discriminability than using the tests alone [40,41]. The final score ranges from 1 to 7, where a higher score indicates higher numeracy.

#### Science literacy

It was assessed with three questions adapted from the US National Science Foundation survey on Science and Engineering Indicators [29]. The three items measure participants’ basic understanding of how science generates evidence (e.g., that a control group is necessary to establish the effectiveness of a treatment). Each item was scored as correct (1) or incorrect (0). The final score was a sum of the correct items (0–3).

### Analysis

We first report descriptive statistics and simple correlations between the measured constructs. The main outcome variables were comprehension and recommendations. Using multiple linear regression analysis (GENLIN command in SPSS) we then investigated what factors uniquely predict comprehension and recommendations. In all analyses we controlled for participants’ gender, stage of training (resident vs. intern/student), and having received statistical education. Finally, following previous models obtained in laypersons [9,17] and based on the correlation results, we conducted path analysis using the Process SPSS macro [42] to investigate how physicians’ beliefs and abilities influenced comprehension, and how comprehension and perceptions of benefits and harms influenced recommendations.

## Results

About half of the participants (N = 89, 51%) reported completing a methods and/or statistics course and only 12 (7%) reported having published a scientific article in an indexed journal. Table 2 shows that, on average, participants had strong positive beliefs about screening, answered about 3 out of 7 numeracy questions correctly, 2 out of 3 science literacy questions, and 2 out of 4 knowledge of screening statistics questions. The comprehension of evidence assessment achieved good discriminability following a normal distribution. Table 1 shows the percentages of correct responses to the individual items.

**Table 2:** Means and standard deviations (SD) of the dependent variables as a function of experimental conditions.

	No demand (N = 86)		Demand (N = 86)		Ineffective (N = 87)		Effective (N = 85)		Total (N = 172)			
	Mean	SD	Mean	SD	Mean	SD	Mean	SD	Mean	SD	Min.	Max.
A-priori screening beliefs	27.90	6.27	28.24	6.38	28.31	6.21	27.84	6.43	28.08	6.30	5	35
Numeracy BNT-Schwarz	2.97	1.56	3.30	1.62	3.38	1.64	2.88	1.52	3.13	1.59	0	7
Numeracy BNT only	0.84	0.92	1.19	1.03	1.17	1.08	0.85	0.87	1.01	0.99	0	4
Numeracy Schwarz only	2.13	0.90	2.11	0.87	2.21	0.86	2.04	0.92	2.12	0.89	0	3
Science literacy	2.08	0.96	2.24	0.91	2.11	0.92	2.21	0.95	2.16	0.93	0	3
Knowledge of screening statistics	1.73	0.85	1.80	0.85	1.82	0.93	1.72	0.75	1.77	0.85	0	4
Comprehension of the evidence	2.64	1.24	2.67	1.18	2.57	1.37	2.74	1.01	2.66	1.21	0	5
Perceived benefits	3.48	1.50	3.58	1.49	3.26	1.72	3.80	1.16	3.53	1.49	1	6
Perceived harms	2.40	1.28	2.74	1.52	2.41	1.46	2.73	1.35	2.57	1.41	1	6
Recommendation	4.00	1.24	4.07	1.26	3.91	1.49	4.16	0.92	4.03	1.25	1	6

Table 3 shows simple correlations between the study variables across all conditions and S1 Table shows these correlations as a function of screening effectiveness. There were three important differences in the correlation patterns between conditions. First, a-priori beliefs about screening were related to stronger screening recommendations in the effective condition only (effective: *r* = .317, *p* = .003 vs. ineffective: *r* = .146, *p* = .177). Numeracy was related to smaller perceived harms (effective: *r* = -.097, *p* = .376 vs. ineffective: *r* = -.232, *p* = .031) and less strong recommendations (effective: *r* = -.139, *p* = .204 vs. ineffective: *r* = -.324, *p* = .002 ) in the ineffective condition only. Finally, comprehension of the evidence was related to fewer perceived benefits *(*effective: *r* = -.014, *p* = .898 vs. ineffective: *r* = .-618, *p* < .001) and less strong recommendations in the ineffective condition only (effective: *r* = -.094, *p* = .393 vs. ineffective: *r* = -.583, *p* < .001).

**Table 3:** Pearson correlations and p values (in parentheses) between the continuous variables.

	Numeracy	Science literacy	Knowledge of screening statistics	Comprehension of the evidence	Perceived benefits	Perceived harms	Recommendation
A-priori screening beliefs	-.103 (.177)	-.065 (.400)	-.209* (.006)	-.116 (.131)	.105 (.170)	.013 (.867)	.200* (.008)
Numeracy		.182* (.017)	.162* (.034)	.219* (.004)	-.121 (.114)	-.185* (.015)	-.264* (< .001)
Science literacy			.033 (.664)	.060 (.433)	-.066 (.388)	-.035 (.646)	-.145 (.057)
Knowledge of screening statistics				.219* (.004)	-.212* (.005)	-.079 (.301)	-.203* (.008)
Comprehension of the evidence					-.399* (< .001)	.012 (.871)	-.420* (< .001)
Perceived benefits						.109 (.156)	.678* (< .001)
Perceived harms							.005 (.945)

* significance according to p < .05.

### What factors predicted comprehension of the evidence?

As shown in Table 4A, in multiple regression analysis, knowledge of screening statistics and numeracy were significant and unique predictors of comprehension. Contrary to our expectation, a-priori beliefs about screening and science literacy were not related to comprehension (see Table 4A for detailed statistical results).

**Table 4:** Multiple linear regression analyses results for the dependent variables comprehension (A), and recommendation (B).

**A: Comprehension**											
**Predictor**	**B**		**SE**			**95% CI**					
						**Inferior**		**Superior**		**Wald Chi**^**2**^	**Sig.**
Intercept	2.26		0.59			1.11		3.41		14.80	< .001
Screening (ineffective vs. effective)	-0.25		0.18			-0.60		0.09		2.03	.155
Patient demand (no demand vs demand)	0.02		0.17			-0.33		0.36		0.01	.931
Gender (male vs. female)	-0.09		0.18			-0.44		0.25		0.28	.598
Experience (intern/student vs. resident)	0.15		0.20			-0.23		0.54		0.61	.436
Statistical education (no vs. yes)	-0.24		0.18			-0.60		0.11		1.81	.178
A-priori screening beliefs	-0.01		0.01			-0.04		0.02		0.46	.497
Numeracy	**0.13**		**0.06**			**0.01**		**0.25**		**4.73**	**.030**
Science literacy	0.01		0.10			-0.17		0.20		0.02	.897
Knowledge of screening statistics	**0.26**		**0.11**			**0.05**		**0.47**		**6.15**	**.013**
**B: Recommendations**											
**Predictor**		**B**		**SE**	**95% CI**						
					**Inferior**		**Superior**		**Wald Chi**^**2**^		**Sig.**
Intercept		4.32		0.57	3.20		5.44		57.21		< .001
Screening (ineffective vs. effective)		**1.26**		**0.38**	**0.51**		**2.01**		**10.76**		**.001**
Patient demand (no demand vs. demand)		-0.03		0.15	-0.32		0.26		0.04		.849
Gender (male vs. female)		-0.27		0.17	-0.60		0.06		2.62		.105
Experience (intern/student vs. resident)		0.24		0.16	-0.07		0.54		2.32		.128
Statistical education (no vs. yes)		**-0.64**		**0.15**	**-0.93**		**-0.34**		**18.01**		**< .001**
A-priori screening beliefs		0.02		0.01	0.00		0.05		3.82		.051
Comprehension		0.01		0.11	-0.20		0.22		0.00		.952
Numeracy		-0.04		0.05	-0.15		0.06		0.71		.401
Knowledge of screening statistics		-0.14		0.08	-0.30		0.02		3.09		.079
Science literacy		-0.07		0.09	-0.25		0.11		0.57		.449
Screening effectiveness*Comprehension		**-0.57**		**0.13**	**-0.83**		**-0.31**		**18.94**		**< .001**

B = unstandardized coefficients, CI = confidence intervals.

### What factors predicted recommendations?

**Fig 2:**
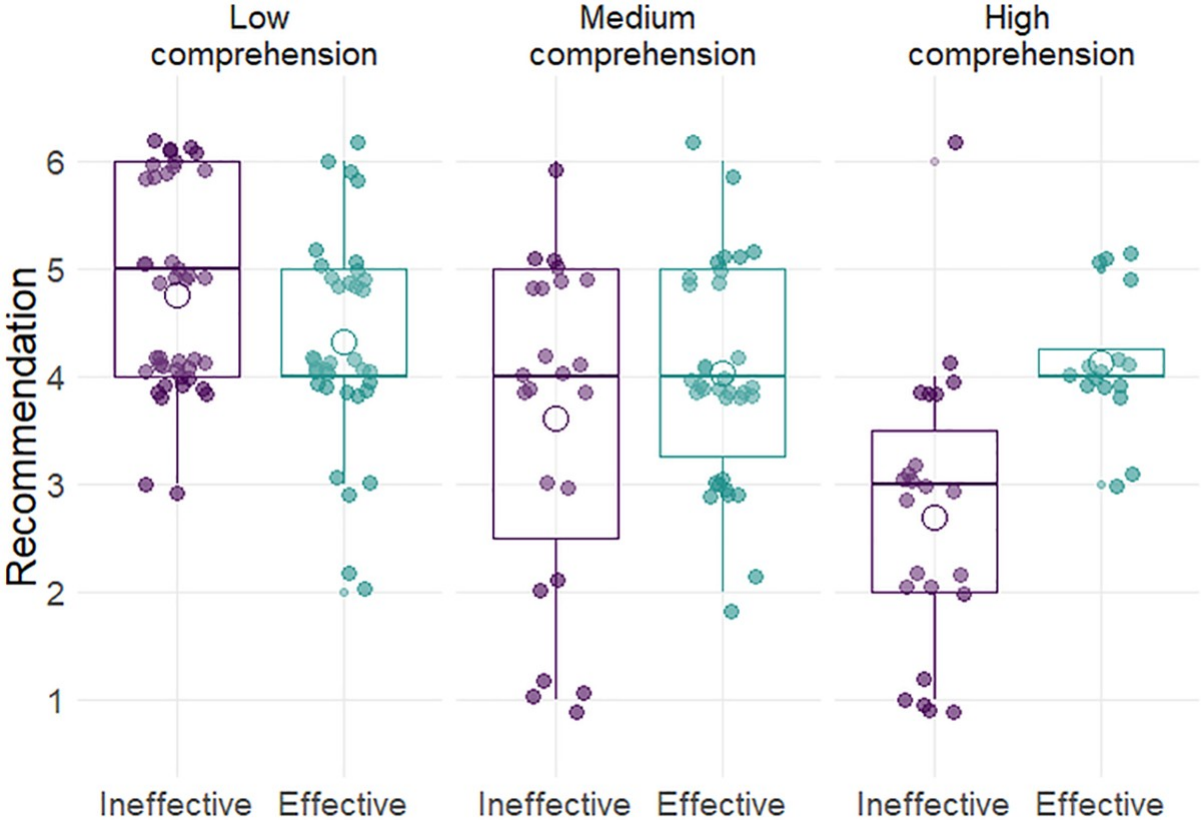
Effect of the screening effectiveness manipulations on recommendations as a function of comprehension. Illustration is based on terciles: low, medium, and high.

As shown in Table 4B, in multiple regression analysis, screening effectiveness had a significant effect on recommendations, which was also qualified by an interaction with comprehension. Fig 2 illustrates that physicians with low levels of comprehension tended to recommend the screening regardless of its effectiveness, being as likely to recommend the effective as the ineffective screening. In contrast, physicians with higher levels of comprehension were influenced by the screening effectiveness and tended to recommend against the ineffective screening. Having received previous statistical education was associated with lower screening recommendations. Contrary to our expectations, patient demand had no effect on recommendations.

Finally, more positive a-priori screening beliefs were marginally associated with stronger recommendations in favor of screening (see Table 4B for statistical details). We had not predicted a difference between conditions in this relationship. However, the correlation results in S1 Table showed that this relationship was only observed in the effective screening condition. Thus, we tested for an interaction between screening beliefs and screening effectiveness but it was not significant, *B* = -.03, *SE* = .02, *p* = .296.

### A decision process model of physicians’ recommendations: path analysis

Next, following previous theoretical models on the effect of beliefs and skills on comprehension, perceptions, and intentions regarding screening participation of patients [9,17], and based on the correlation results in Table 2 we tested a path model with main outcome screening recommendations. This model tested whether a-priori screening beliefs and numeracy predict knowledge of screening statistics, which in turn predicts comprehension, perceived benefits, perceived harms, and recommendations. To estimate indirect effects we fitted model 6 from the SPSS Process Marco [42] and computed 95% confidence intervals (CI) based on 5000 bootstrap samples. We also entered the effects of the experimental manipulations and the demographic and experience variables.

**Fig 3:**
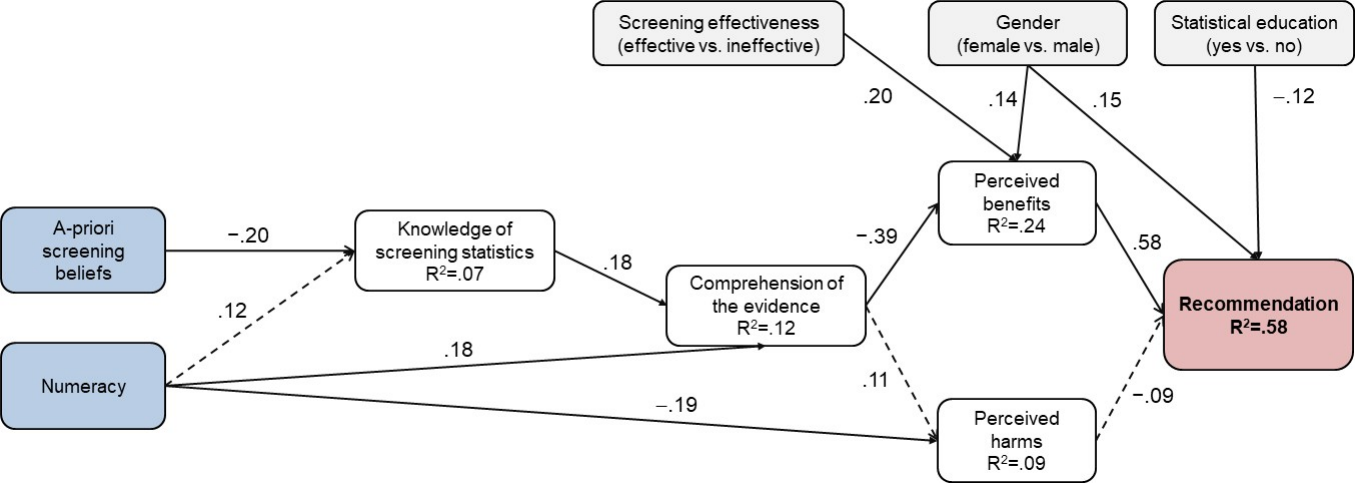
Path analysis results. Displayed coefficients are standardized Betas. Continuous lines indicate significant paths (*p* < .05). Dashed lines indicate non-significant paths (*p*>.05) that were hypothesized to be significant. R^2^ = percentage of explained variance by all predictors. Blue indicates independent variables, white mediator variables, grey control variables, and red the outcome variable.

The main results are displayed in Fig 3 and detailed results of the regressions underlying the indirect effects are available in S1 File. There were significant indirect effects (i) from a-priori screening beliefs on recommendations via knowledge of screening statistics, comprehension, and perceived benefits, unstandardized effect (*UE*) = .002, 95% CI [.0003, .005], and (ii) from numeracy on recommendations via comprehension and perceived benefits, *UE* = −.031, 95% CI [−.075, −.003]. Physicians with higher numeracy and less positive screening beliefs had better comprehension, perceived less benefits from screening, and were less likely to recommend it; perceived harms were not significant predictors of recommendations. The effect of positive screening beliefs in particular was further mediated by knowledge of screening statistics (see Fig 3).

Fig 3 also displays the significant effects of the other variables in the model. More benefits were perceived in the effective compared to the ineffective condition and females perceived more benefit from screening compared to males. Higher numeracy was related to less perceived harm, and finally, females and those who had not received previous statistical education made stronger screening recommendations.

Related to the above-mentioned effect of numeracy and despite the lack of significant relationship between perceived harms and recommendations, there was also an indirect effect of numeracy on recommendations via perceived harms, *UE* = .014, 95% CI [.0001, .044]; however, indirect effects contrasts showed that it was negligible compared to the effect via perceived benefits, contrast *UE* = −.045, 95% CI [−.095, −.012]. An examination of the differences in perceived harms showed that participants with higher numeracy (highest tercile) tended to rate harms “very small” whereas participants with lower numeracy (medium and lowest tercile) rated harms as “small”.

## Discussion

This study demonstrated the importance of understanding evidence about screening effectiveness for preventing biased and misleading physician recommendations. It also identified (a) a-priori positive beliefs about screening as markers of low cancer screening risk literacy, (b) physician numeracy as a specific skill that can foster comprehension and help counteract biases, and (c) knowledge of screening statistics as specific knowledge required for the correct evaluation of screening effectiveness.

### Comprehension

We found similar comprehension and knowledge gaps as those documented in previous research with physicians in training and experienced physicians in other countries (i.e., Germany, USA, UK) [10,13,43]. Many of the participants surveyed had difficulties understanding and interpreting important statistics used to evaluate and communicate the effectiveness of cancer screening (see Table 1). Only 50% of physicians in training could correctly deduce if a screening test saved lives based on detection and mortality data from a 10-year-long trial. Consistent with research in practicing experienced physicians [10,13], even fewer physicians in training knew that if screening is associated with improved survival rates it does not necessarily mean that it is life-saving. This is an important problem because, unfortunately, survival rates are sometimes used to promote screening (e.g., [44]).

Fortunately, a recent study showed that even 90 minutes of training can dramatically improve the risk literacy of medical professionals in training (i.e., from median 50% to 90% correct on a basic medical literacy test) [43]. The training in question included evidence-based strategies that have been shown to improve comprehension such as the design of facts boxes and natural frequency trees [43]. Another example for effective strategies are visual aids (for a review see [45]). For instance, in a recent study with surgeons, a simple visual aid in the form of icon arrays increased deliberation time and improved risk interpretation [22].

### Beliefs about screening

Positive a-priori beliefs about screening were related to stronger recommendations in favor of screening via knowledge of screening statistics and comprehension of the evidence. In other words, physicians who tended to view screenings very positively were not aware of what statistics should be consulted to rate screening effectiveness, which contributed to their lower comprehension of the presented evidence and stronger recommendations in favor of screening. This suggests that participants who already had a strong positive opinion about the value of screening in general may have been less likely to examine the statistical questions and evidence critically and thoroughly, leading to wrong answers. Another possible explanation is that participants with stronger positive beliefs were previously exposed to misleading statistics regarding screening or were never exposed to information about screenings with little or no effectiveness. This could have helped generate their strong positive beliefs about screenings and contributed to their inability to properly evaluate the evidence presented. Whatever the mechanisms, making recommendations to patients based on general beliefs when the evidence at hand is at odds with these beliefs represents a bias, and the extensive training received did not protect the physicians in our study from such misguided judgments that are also found in laypersons [17]. Future research should investigate if the strength of this bias increases with practice and experience or fades away.

### Specific physician competencies

The specific knowledge about what screening statistics are relevant to assess screening effectiveness was a unique predictor of correct comprehension of the evidence, suggesting that this knowledge should be part of medical curricula for specialties where screenings for diseases such as cancer are relevant.

In addition, consistent with previous research in experienced physicians [24], high numeracy was related to an increase in comprehension that was independent of all other assessed factors. These results are also in line with a recent study that showed that practicing physicians with lower (vs. higher) numeracy were more likely to offer incomplete and misleading communication about cancer screening to a hypothetical patient [13]. The current results, together with emerging literature of risk literacy in physicians [1,10,22,23,46,47], suggests that numeracy is a major building block of medical professionals’ risk literacy, risk communication skill, and decision making expertise, with benefits easily transferable across settings [4]. This means that emphasizing statistical numeracy in medical curricula and continuing education may not only help physicians understand screening statistics but is likely to have benefits for understanding and risk communication across diverse contexts [4].

### A decision process model of physicians’ recommendations

A process model similar to that found in laypersons [9,17] showed that comprehension and perceived benefits from screening were central to physicians’ recommendations to the hypothetical patient. Whereas physicians with high levels of comprehension were risk literate decision makers–they were likely to slightly recommend the effective screening and recommend against the ineffective screening, physicians with medium and low levels of comprehension were about equally likely to recommend both screenings (see Fig 3). This result directly demonstrates the importance of comprehension of screening statistics to prevent misleading and potentially harmful physician recommendations. The obtained process model further showed that biased recommendations were due to, on one hand, physicians’ already existing beliefs about the goodness of screening, which may have guided their evaluation of the evidence, and on the other hand, physicians’ low numeracy (Fig 3).

Also similar to results obtained in laypersons, perceived harms were much less predictive of decisions [17]. It is possible that in the context of prevention and early detection benefits generally receive more weight than harms [17]. However, this should be investigated in more detail because in the current study little emphasis was placed on harms (i.e., harms from overdiagnosis were not directly discussed or quantified but had to be inferred, and false positive tests were not mentioned) and the perceptions of harm were generally very low, which may be the reason why they did not emerge as a significant predictor of recommendations.

### Limitations and future directions

Whereas the current results show that numeracy and better statistical knowledge can help counteract the detrimental effects of previous beliefs, science literacy did not emerge as an important factor. However, the scale used in the current research was brief and also easy for the surveyed population. Future research should investigate the role of science literacy using more appropriate and elaborate instruments.

Similarly, the patient demand manipulation did not show the expected effect on recommendations. On one hand, it is possible that the manipulation was not strong enough to produce an effect–in the demand condition the patient was said to want to undergo the screening but nevertheless requested the physician’s opinion. On the other hand, patient demand may not be important among inexperienced physicians in artificial scenarios. However, it is often mentioned by physicians themselves as a determining factor and thus is likely highly important in real clinical situations [33–37].

Participants of the current study were enrolled in one of the top-ranking medical programs in Peru and in Latin America. Given that cross-cultural differences in risk literacy have been documented [25], it is not clear to what extent results from this sample of students will fully generalize to other samples or to actual recommendations of experienced, practicing physicians. Nevertheless, the current results, together with previous findings in diverse populations, suggest that gaps in comprehension are common and their detrimental effects on communication and decisions are robust [9,10,13].

## Conclusion

Despite a rich literature on patient risk literacy, not many studies have addressed what influences physicians’ risk literacy. Given the multiple nuances and challenges of doctor-patient communication, research on physician risk literacy beyond artificial scenarios and in actual interaction with patients is needed. The current results demonstrate that in the context of cancer screening, a-priori positive beliefs about the goodness and desirability of screening, likely reinforced by multiple screening campaigns, and low physician numeracy can be important precursors of low physician risk literacy and biased, misleading recommendations.

## Supporting information

S1 Table Pearson correlations and p values (in parentheses, * significance according to p < .05) between the continuous variables as a function of screening effectiveness. (DOCX)

S1 File Detailed results of the regressions underlying the indirect effects. (PDF)
