# Supplementary material for: Cancer screening risk literacy of physicians in training: An experimental study
Source: PLoS One. 2019 Jul 3;14(7):e0218821. doi: 10.1371/journal.pone.0218821 (PMC6608976; doi:10.1371/journal.pone.0218821)
Supplement: S1 Table — (DOCX) [file pone.0218821.s001.docx]

| **A: Screening=ineffective, N=87** | Numeracy | Science literacy | Knowledge of screening statistics | Comprehension of the evidence | Perceived benefits | Perceived harms | Recommendation |
| --- | --- | --- | --- | --- | --- | --- | --- |
| Screening beliefs | -.134 | -.073 | -.210 | -.114 | .085 | -.054 | .146 |
|  | (.216) | (.499) | (.050) | (.292) | (.436) | (.619) | (.177) |
| Numeracy |  | .241^*^ | .175 | .208 | -.205 | -.232^*^ | -.324^**^ |
|  |  | (.025) | (.104) | (.054) | (.057) | (.031) | (.002) |
| Science literacy |  |  | .201 | .085 | -.196 | -.105 | -.196 |
|  |  |  | (.062) | (.432) | (.070) | (.333) | (.070) |
| Knowledge of screening statistics |  |  |  | .247^*^ | -.194 | -.182 | -.204 |
|  |  |  |  | (.021) | (.072) | (.091) | (.058) |
| Comprehension of the evidence |  |  |  |  | -.618^**^ | .008 | -.583^**^ |
|  |  |  |  |  | (.000) | (.944) | (.000) |
| Perceived benefits |  |  |  |  |  | .081 | .689^**^ |
|  |  |  |  |  |  | (.456) | (.000) |
| Perceived harms |  |  |  |  |  |  | -.041 |
|  |  |  |  |  |  |  | (.706) |

**Table S1. Pearson correlations and p values (in parentheses, * significance according to p<.05) between the continuous variables as a function of screening effectiveness.**

| **B: Screening=effective, N=85** | Numeracy | Science literacy | Knowledge of screen. stats | Comprehension evidence | Perceived benefits | Perceived harms | Recommendation |
| --- | --- | --- | --- | --- | --- | --- | --- |
| Screening beliefs | -.086 | -.053 | -.217^*^ | -.116 | .163 | .094 | .317^**^ |
|  | (.432) | (.633) | (.046) | (.289) | (.137) | (.394) | (.003) |
| Numeracy |  | .141 | .128 | .275^*^ | .081 | -.097 | -.139 |
|  |  | (.197) | (.245) | (.011) | (.461) | (.376) | (.204) |
| Science literacy |  |  | -.165 | .020 | .092 | .027 | -.094 |
|  |  |  | (.130) | (.853) | (.400) | (.809) | (.391) |
| Knowledge of screening statistics |  |  |  | .185 | -.229^*^ | .077 | -.190 |
|  |  |  |  | (.091) | (.035) | (.486) | (.082) |
| Comprehension of the evidence |  |  |  |  | -.014 | .000 | -.094 |
|  |  |  |  |  | (.898) | (.997) | (.393) |
| Perceived benefits |  |  |  |  |  | .109 | .640^**^ |
|  |  |  |  |  |  | (.319) | (.000) |
| Perceived harms |  |  |  |  |  |  | .055 |
|  |  |  |  |  |  |  | (.615) |
