# Supplementary material for: Cancer screening risk literacy of physicians in training: An experimental study
Source: PLoS One. 2019 Jul 3;14(7):e0218821. doi: 10.1371/journal.pone.0218821 (PMC6608976; doi:10.1371/journal.pone.0218821)
Supplement: S1 File — (PDF) [file pone.0218821.s002.pdf]

Variable key:

recommend=recommendation
screenbe=screening beliefs
knowlR1=knowledge of screening statistics
compR1=comprehension
benefits=perceived benefits
harms=perceived harms
numeracy=numeracy
effective=Screening effectiveness (1=effective, 0=ineffective)
demand= Demand for screening (1=demand, 0=no demand)
gender=Gender (1=female, 0=male)
resident=Resident (1=resident, 0=other)
statsed=Statistical education (1=yes, 0=no)
Significant effects are marked in red

***Indirect effects for screening beliefs**

```
PROCESS vars =numeracy screenbeliefs compR1 knowlR1 recommendation harms benefits
effective demand gender resident statsed/y= recommendation/x=screenbeliefs /m=
knowlR1 compR1 benefits harms/model=6/conf=95/boot=5000/contrast=1.

Run MATRIX procedure:

***************** PROCESS Procedure for SPSS Release 2.11 ****************

          Written by Andrew F. Hayes, Ph.D.       www.afhayes.com
    Documentation available in Hayes (2013). www.guilford.com/p/hayes3

**************************************************************************
Model = 6
    Y = recommend=recommendation
    X = screenbe=screening beliefs
   M1 = knowlR1=knowledge of screening statistics
   M2 = compR1=comprehension
   M3 = benefits=perceived benefits
   M4 = harms=perceived harms

Statistical Controls:
CONTROL= numeracy effectiv demand   gender   resident statsed

Sample size
        172

**************************************************************************

******************** DIRECT AND INDIRECT EFFECTS *************************

Direct effect of X on Y
     Effect         SE          t          p       LLCI       ULCI
      .0186      .0105     1.7764      .0776     -.0021      .0393

Indirect effect(s) of X on Y
           Effect    Boot SE   BootLLCI   BootULCI
Total:      .0132      .0110     -.0072      .0364
Ind1 :      .0002      .0024     -.0045      .0054
```

```
Ind2 :       .0009      .0007      .0001      .0034
Ind3 :       .0023      .0018     -.0004      .0072
Ind4 :      -.0002      .0004     -.0016      .0002
Ind5 :       .0016      .0010      .0003      .0046
Ind6 :       .0001      .0001      .0000      .0005
Ind7 :       .0000      .0001     -.0003      .0000
Ind8 :       .0000      .0000     -.0002      .0000
Ind9 :       .0012      .0020     -.0017      .0066
Ind10:       .0023      .0034     -.0038      .0097
Ind11:       .0001      .0002     -.0001      .0011
Ind12:       .0000      .0001     -.0005      .0000
Ind13:       .0046      .0090     -.0124      .0229
Ind14:      -.0001      .0003     -.0013      .0001
Ind15:       .0004      .0019     -.0023      .0060
(C17)       -.0008      .0008     -.0036      .0001

Indirect effect key
 Ind1 :   screenbe ->       knowlR1   ->       recommen
 Ind2 :   screenbe ->       knowlR1   ->       compR1    ->       recommen
 Ind3 :   screenbe ->       knowlR1   ->       benefits ->       recommen
 Ind4 :   screenbe ->       knowlR1   ->       harms     ->       recommen
 Ind5 :   screenbe ->       knowlR1   ->       compR1    ->       benefits ->
recommen
 Ind6 :   screenbe ->       knowlR1   ->       compR1    ->       harms     ->
recommen
 Ind7 :   screenbe ->       knowlR1   ->       benefits ->       harms     ->
recommen
 Ind8 :   screenbe ->       knowlR1   ->       compR1    ->       benefits ->
harms     ->       recommen
 Ind9 :   screenbe ->       compR1    ->       recommen
 Ind10:   screenbe ->       compR1    ->       benefits ->       recommen
 Ind11:   screenbe ->       compR1    ->       harms     ->       recommen
 Ind12:   screenbe ->       compR1    ->       benefits ->       harms     ->
recommen
 Ind13:   screenbe ->       benefits ->       recommen
 Ind14:   screenbe ->       benefits ->       harms     ->       recommen
 Ind15:   screenbe ->       harms     ->       recommen

Specific indirect effect contrast definitions
(C17)     Ind2       minus      Ind5

******************** ANALYSIS NOTES AND WARNINGS *************************

Number of bootstrap samples for bias corrected bootstrap confidence intervals:
     5000

Level of confidence for all confidence intervals in output:
    95.00

------ END MATRIX -----
```

*Indirect effects for numeracy

```
PROCESS vars =numeracy screenbeliefs compR1 knowlR1 recommendation harms benefits
effective demand gender resident statsed/y= recommendation/x=numeracy /m= knowlR1
compR1 benefits harms/model=6/conf=95/boot=5000/contrast=1.


Run MATRIX procedure:

***************** PROCESS Procedure for SPSS Release 2.11 ****************

          Written by Andrew F. Hayes, Ph.D.       www.afhayes.com
    Documentation available in Hayes (2013). www.guilford.com/p/hayes3

**************************************************************************
Model = 6
    Y = recommen
    X = numeracy
   M1 = knowlR1
   M2 = compR1
   M3 = benefits
   M4 = harms

Statistical Controls:
CONTROL= screenbe effectiv demand   gender   resident statsed

Sample size
        172

******************** DIRECT AND INDIRECT EFFECTS *************************

Direct effect of X on Y
     Effect         SE          t          p       LLCI       ULCI
     -.0821      .0453    -1.8144      .0715     -.1715      .0073

Indirect effect(s) of X on Y
           Effect    Boot SE   BootLLCI   BootULCI
Total:     -.0300      .0417     -.1170      .0454
Ind1 :     -.0005      .0069     -.0195      .0116
Ind2 :     -.0021      .0021     -.0099      .0001
Ind3 :     -.0057      .0064     -.0267      .0013
Ind4 :      .0005      .0012     -.0004      .0061
Ind5 :     -.0040      .0038     -.0157      .0004
Ind6 :     -.0002      .0003     -.0017      .0000
Ind7 :      .0001      .0002      .0000      .0012
Ind8 :      .0001      .0001      .0000      .0008
Ind9 :     -.0165      .0127     -.0553     -.0006
Ind10:     -.0313      .0176     -.0747     -.0034
Ind11:     -.0013      .0018     -.0089      .0001
Ind12:      .0006      .0007      .0000      .0037
Ind13:      .0170      .0334     -.0483      .0846
Ind14:     -.0003      .0009     -.0041      .0005
Ind15:      .0136      .0101      .0001      .0441
(C85)       .0147      .0145     -.0033      .0581
(C95)      -.0449      .0202     -.0946     -.0123
```

```
Indirect effect key
 Ind1 :   numeracy ->       knowlR1   ->       recommen
 Ind2 :   numeracy ->       knowlR1   ->       compR1    ->       recommen
 Ind3 :   numeracy ->       knowlR1   ->       benefits ->       recommen
 Ind4 :   numeracy ->       knowlR1   ->       harms     ->       recommen
 Ind5 :   numeracy ->       knowlR1   ->       compR1    ->       benefits ->
recommen
 Ind6 :   numeracy ->       knowlR1   ->       compR1    ->       harms     ->
recommen
 Ind7 :   numeracy ->       knowlR1   ->       benefits ->       harms     ->
recommen
 Ind8 :   numeracy ->       knowlR1   ->       compR1    ->       benefits ->
harms     ->       recommen
 Ind9 :   numeracy ->       compR1    ->       recommen
 Ind10:   numeracy ->       compR1    ->       benefits ->       recommen
 Ind11:   numeracy ->       compR1    ->       harms     ->       recommen
 Ind12:   numeracy ->       compR1    ->       benefits ->       harms     ->
recommen
 Ind13:   numeracy ->       benefits ->       recommen
 Ind14:   numeracy ->       benefits ->       harms     ->       recommen
 Ind15:   numeracy ->       harms     ->       recommen

Specific indirect effect contrast definitions
(C85)    Ind9       minus      Ind10
(C95)    Ind10      minus      Ind15

******************** ANALYSIS NOTES AND WARNINGS *************************

Number of bootstrap samples for bias corrected bootstrap confidence intervals:
     5000

Level of confidence for all confidence intervals in output:
    95.00

------ END MATRIX -----
```

*Detailed results from the underlying multiple regressions

Outcome: knowlR1

Model Summary

R	R-sq	F	df1	df2	p
.2619	.0686	1.7252	7.0000	164.0000	.1064

Model

	coeff	se	t	p	LLCI	ULCI
constant	2.3702	.3821	6.2028	.0000	1.6157	3.1247
screenbe	-.0262	.0102	-2.5666	.0112	-.0464	-.0061
numeracy	.0647	.0434	1.4888	.1385	-.0211	.1504
effectiv	-.0741	.1293	-.5732	.5673	-.3294	.1812
demand	.0555	.1284	.4324	.6660	-.1979	.3089
gender	-.0761	.1302	-.5844	.5597	-.3332	.1810
resident	-.0190	.1454	-.1306	.8963	-.3060	.2680
statsed	-.0130	.1330	-.0979	.9221	-.2755	.2495

****

Outcome: compR1

Model Summary

R	R-sq	F	df1	df2	p
.3389	.1149	2.6439	8.0000	163.0000	.0094

Model

	coeff	se	t	p	LLCI	ULCI
constant	1.8601	.5912	3.1466	.0020	.6928	3.0274
knowlR1	.2624	.1087	2.4134	.0169	.0477	.4771
screenbe	-.0097	.0145	-.6685	.5048	-.0384	.0190
numeracy	.1328	.0609	2.1814	.0306	.0126	.2530
effectiv	.2524	.1802	1.4009	.1631	-.1034	.6082
demand	-.0135	.1788	-.0754	.9400	-.3666	.3396
gender	.0946	.1815	.5212	.6029	-.2638	.4529
resident	-.1518	.2024	-.7501	.4543	-.5515	.2478
statsed	.2439	.1851	1.3177	.1895	-.1216	.6095

****

Outcome: benefits

Model Summary

R	R-sq	F	df1	df2	p
.4884	.2385	5.6387	9.0000	162.0000	.0000

Model

	coeff	se	t	p	LLCI	ULCI
constant	4.2606	.7009	6.0789	.0000	2.8765	5.6446
knowlR1	-.1809	.1274	-1.4199	.1576	-.4324	.0707
compR1	-.4865	.0902	-5.3951	.0000	-.6645	-.3084
screenbe	.0095	.0167	.5692	.5700	-.0235	.0426
numeracy	.0352	.0711	.4953	.6210	-.1052	.1756
effectiv	.5964	.2087	2.8579	.0048	.1843	1.0084
demand	.1243	.2058	.6038	.5468	-.2822	.5308
gender	.4135	.2091	1.9775	.0497	.0006	.8263
resident	-.0639	.2334	-.2738	.7846	-.5248	.3970

statsed	-.0515	.2142	-.2402	.8105	-.4745	.3716

****

Outcome: harms

Model Summary

R	R-sq	F	df1	df2	p
.2969	.0881	1.5561	10.0000	161.0000	.1243

Model

	coeff	se	t	p	LLCI	ULCI
constant	2.2407	.8058	2.7808	.0061	.6494	3.8319
knowlR1	-.0938	.1330	-.7056	.4814	-.3564	.1687
compR1	.1269	.1016	1.2495	.2133	-.0737	.3276
benefits	.1077	.0815	1.3214	.1883	-.0533	.2687
screenbe	-.0048	.0174	-.2776	.7817	-.0392	.0295
numeracy	-.1715	.0738	-2.3240	.0214	-.3173	-.0258
effectiv	.1601	.2219	.7217	.4715	-.2780	.5983
demand	.4059	.2138	1.8987	.0594	-.0163	.8281
gender	-.1735	.2195	-.7905	.4304	-.6070	.2600
resident	.2718	.2422	1.1225	.2633	-.2064	.7501
statsed	.1406	.2223	.6323	.5281	-.2984	.5796

****

Outcome: recommen

Model Summary

R	R-sq	F	df1	df2	p
.7603	.5780	19.9255	11.0000	160.0000	.0000

Model

	coeff	se	t	p	LLCI	ULCI
constant	2.3832	.4976	4.7898	.0000	1.4006	3.3659
knowlR1	-.0074	.0803	-.0924	.9265	-.1661	.1512
compR1	-.1244	.0616	-2.0205	.0450	-.2460	-.0028
benefits	.4839	.0494	9.7889	.0000	.3862	.5815
harms	-.0794	.0475	-1.6706	.0967	-.1733	.0145
screenbe	.0186	.0105	1.7764	.0776	-.0021	.0393
numeracy	-.0821	.0453	-1.8144	.0715	-.1715	.0073
effectiv	-.0165	.1340	-.1230	.9023	-.2812	.2482
demand	.0623	.1304	.4780	.6333	-.1952	.3198
gender	.3842	.1327	2.8961	.0043	.1222	.6462
resident	.2318	.1467	1.5805	.1160	-.0578	.5214
statsed	-.3033	.1343	-2.2591	.0252	-.5684	-.0382
